# Mechanisms of Diapause in Major Crustacean Taxa: Environmental Cues, Gene Regulation, and Adaptive Strategies

**DOI:** 10.3390/biology15181587

**Published:** 2026-09-09

**Authors:** Malik Qammar, Atiq Irish, Syeda Maira Hamid, Cheng Ma, Jiawei Xu, Zhichao Wang

**Affiliations:** 1 State Key Laboratory Incubation Base for Conservation and Utilization of Bio-Resource in Tarim Basin, College of Life Science and Technology, Tarim University, Alar 843300, China; qammarmalik24@gmail.com (M.Q.); iatiq@gudgk.edu.pk (A.I.); smairahamid@gmail.com (S.M.H.); chengm0428@163.com (C.M.); 15165235877@163.com (J.X.); 2 Department of Zoology, Ghazi University, Dera Ghazi Khan 32200, Pakistan

**Keywords:** crustacean diapause, metabolic suppression, stress tolerance, FoxO signaling, epigenetic regulation, environmental cues, population resilience

## Abstract

Diapause serves as an adaptive dormancy strategy in freshwater, hypersaline, and marine crustaceans, allowing them to survive harsh conditions like temperature extremes, salinity fluctuations, hypoxia, and food scarcity. This review focuses on diapause across major crustacean taxa, with particular emphasis on branchiopods and copepods. However, detailed mechanistic evidence is more prevalent in well-studied species like *Artemia*, *Daphnia*, and calanoid copepods, leading to an uneven distribution of evidence across these taxa. Thus, the molecular and physiological mechanisms discussed in this review should not be assumed to be universally conserved across Crustacea, and taxon-specific differences and current knowledge gaps are highlighted where appropriate. The review consolidates the current understanding of crustacean diapause, focusing on environmental cues, gene-regulatory networks, hormonal and epigenetic control, metabolic suppression, and stress resilience. In the best-studied taxa, diapause has been associated with changes in the expression and/or activity of antioxidant defenses, small RNAs, energy-storage pathways, heat shock proteins, and FoxO-associated signaling components; however, the evidence for these mechanisms varies among taxa, and their conservation across Crustacea, remains uncertain. The functions of these responses include developmental arrest, energy conservation, cellular protection, and maintaining viability during dormancy. Their significance varies among different groups of organisms and remains incompletely understood in many cases. Diapause also contributes to the persistence of populations by forming dormant propagule banks, coordinating seasonal processes, linking trophic levels, and increasing resilience to environmental changes. The ecological effects of diapause depend on the life stage and habitat. Recent progress in transcriptomics, epigenomics, long-read sequencing, single-cell methodologies, and multi-omics integration present new opportunities for comparing regulatory responses across species. Molecular data primarily focuses on *Artemia*, *Daphnia*, and a limited number of copepod species, posing challenges in generalizing to less represented lineages. Previous syntheses have concentrated on individual taxa, physiological dormancy, or specific molecular mechanisms. This review aims to fill this gap by integrating environmental regulation, taxonomic and life-stage contexts, molecular and physiological mechanisms, and ecological and evolutionary implications systematically. This comparative approach distinguishes between shared regulatory features across species and lineage-specific characteristics, while considering variations in the strength of supporting evidence. Future research should prioritize functional validation, broader taxonomic inclusivity, comparative multi-species analyses, multifactorial experiments, and long-term ecological investigations to enhance understanding of crustacean diapause evolution and refine predictions amidst climate-induced environmental shifts.

## Simple Summary

Diapause is a regulated dormancy state utilized by numerous crustaceans to survive harsh environmental conditions such as salinity fluctuations, food scarcity, and extreme temperatures. During diapause, both development and metabolism are minimized until favorable conditions reemerge. This overview summarizes the current understanding of the environmental cues governing diapause, molecular and physiological mechanisms involved, and variations among the primary crustacean groups. It also assesses recent advancements in genomic, transcriptomic, and other molecular techniques that have enriched our comprehension of diapause regulation, while highlighting significant knowledge gaps that require attention. Enhancing our knowledge of crustacean diapause will not only aid in understanding their adaptation to environmental changes but also prove beneficial in biodiversity conservation, aquaculture, and ecosystem management.

## 1. Introduction

Diapause is an adaptive dormancy strategy, enables numerous crustaceans to survive harsh environmental conditions like temperature extremes, salinity fluctuations, hypoxia, and food scarcity [1,2]. It differs from short-term quiescence, which typically occurs in direct response to adverse conditions, by involving regulated developmental arrest that can either anticipate or endure recurring environmental stress. This strategy is observed in hypersaline, freshwater, and marine crustaceans and can manifest during various life stages, including embryonic, larval, juvenile, or adult phases [3]. The adaptive response of diapause in Crustacea is not consistent due to variations in timing, duration, environmental sensitivity, and ecological function across different lineages and life-history strategies. Freshwater taxa like *Daphnia* often creates resting eggs for diapause that can endure in sediment. On the other hand, *Artemia* forms resistant dormant cysts in unfavorable conditions [4,5,6]. Pelagic copepods, like *Calanus finmarchicus*, often use copepodite-stage diapause to synchronize life-cycle progression with seasonal food availability, annual physiological rhythms, and pelagic ecosystem dynamics [7]. These different approaches demonstrate the variability of crustacean diapause based on environmental predictability, developmental stage, reproductive strategy, and energetic needs.

**Figure 1:**
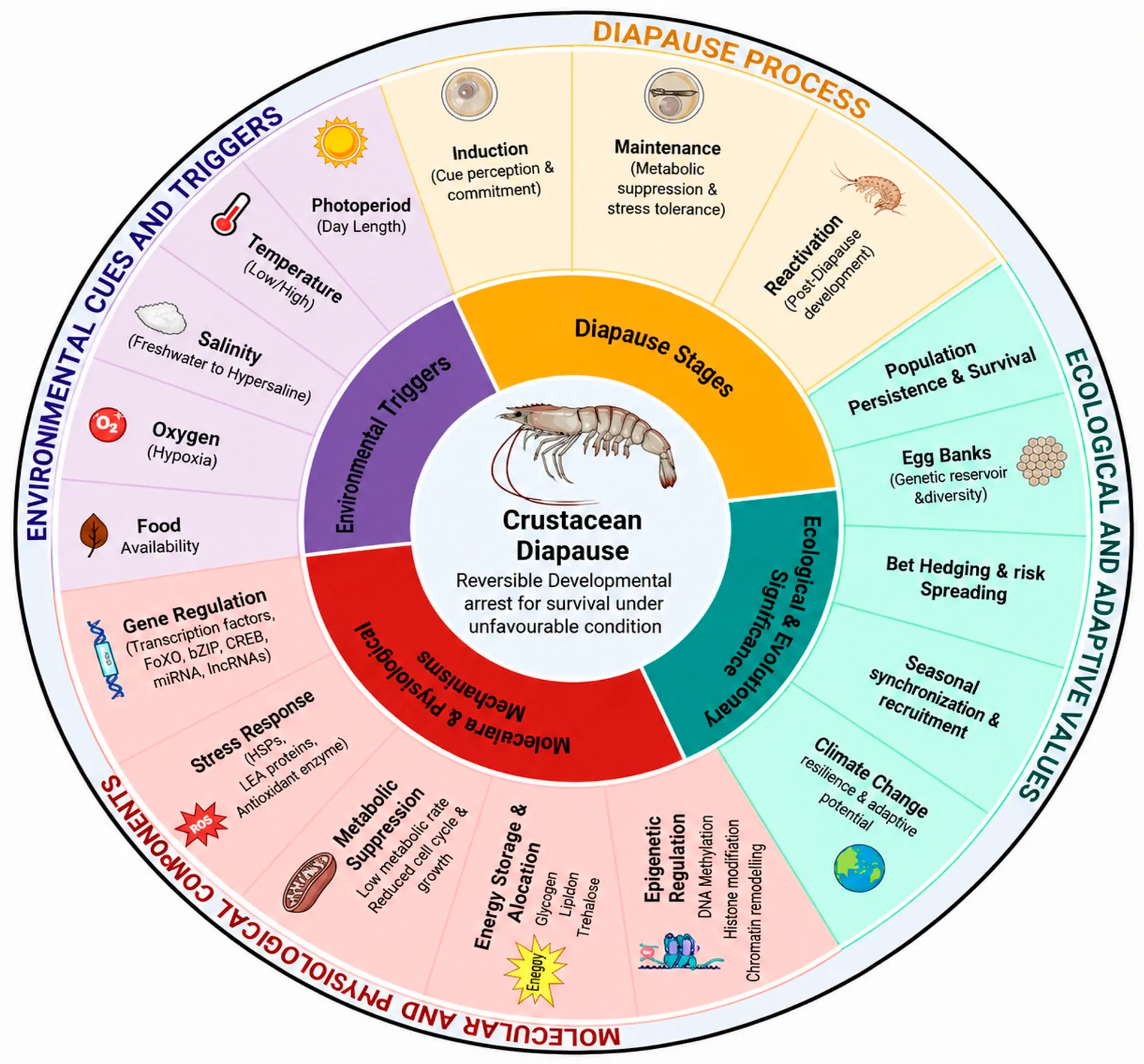
Conceptual overview of crustacean diapause: stages, environmental triggers, molecular and physiological mechanism and adaptive significance.

The regulation of crustacean diapause is a complex interaction of environmental signals, molecular pathways, and physiology. Morphological adaptations of dormancy are accompanied by changes in cell physiology, including the involvement of molecular chaperones (heat shock proteins), antioxidant systems, stress-response pathways, and other mechanisms for the maintenance of cells [4,8]. Moreover, FoxO-dependent signaling, hormonal control, and transcriptional regulatory networks connect environmental perception to metabolic and developmental control. However, these mechanisms are not expressed uniformly across crustaceans. Embryonic diapause in branchiopods is significantly different from copepodite-stage diapause in marine copepods, demonstrating that similar regulatory features may be influenced by species-specific life history and habitat conditions [7,9]. Recent progress in transcriptomics, long-read sequencing, single-cell studies, epigenomics, and multi-omics integration have provided unparalleled opportunities to explore the regulation of diapause across all scales of biology, but these advances lack comparative analyses and functional validation of potential pathways. This review, therefore, highlights the current understanding of environmental, molecular, physiological, and ecological mechanisms involved in crustacean diapause and focuses on interactions among environmental signals, gene-regulatory networks, hormonal signaling, epigenetic modification, and metabolic adaptations (Figure 1).

Previous reviews on diapause have primarily concentrated on specific taxonomic groups or aspects of dormancy, such as physiological stress tolerance, molecular regulation, or life-history responses. However, there has been a lack of focus on integrating environmental cue perception, diapause life stage, molecular and physiological regulation, and ecological consequences in freshwater, hypersaline, and marine crustaceans. Another knowledge gap is the varying strength of available evidence, with mechanisms from functional experiments, physiological studies, and omics-based associations often being discussed together, making it challenging to differentiate broadly conserved mechanisms from lineage-specific adaptations. This review aims to address these gaps by establishing comparative connections among environmental drivers, endocrine and molecular regulation, physiological responses, life-history context, population persistence, and ecosystem-level consequences, while considering the strengths and limitations of the existing evidence.

Recent advances in transcriptomics, long-read sequencing, single-cell approaches, epigenomics, and multi-omics integration have provided new opportunities to investigate diapause across multiple levels of biological organization. However, mechanistic evidence remains uneven across crustacean taxa, and many proposed pathways are supported primarily by transcriptomic or other correlative evidence rather than direct functional validation. Comparative analyses across species, life stages, and habitats are therefore needed to distinguish potentially conserved components of diapause regulation from lineage-specific adaptations and to identify mechanisms requiring further experimental validation.

A unified framework is proposed to organize the review around four interconnected levels: (i) environmental regulation, including photoperiod, temperature, salinity, oxygen availability, food conditions, and multiple stressors; (ii) biological context, including taxonomic group, habitat, and diapause life stage; (iii) molecular and physiological regulation, including gene-expression networks, hormonal and epigenetic control, energy storage, metabolic suppression, and cellular stress protection; and (iv) adaptive consequences, including dormant-stage survival, population persistence, seasonal synchronization, evolutionary diversification, and responses to environmental change. These levels are considered interconnected rather than strictly linear, because environmental conditions can simultaneously influence multiple regulatory pathways, while physiological and metabolic states can affect diapause maintenance and reactivation. Representative taxa were selected based on the availability of published evidence and their contrasting habitats, life stages, and life-history strategies, with particular emphasis on well-studied systems such as *Artemia*, *Daphnia*, and calanoid copepods.

The remainder of the review follows this framework, examining diapause diversity and ecological significance, induction, maintenance, duration, termination, environmental cues, and molecular, hormonal, epigenetic, physiological, and metabolic regulation. Where possible, functional and experimental evidence is distinguished from transcriptomic, proteomic, observational, and comparative associations. The review then considers population-level, ecological, evolutionary, and climate-related consequences and identifies major taxonomic, methodological, functional-validation, and ecological-scaling gaps. This approach facilitates comparisons among crustacean groups without assuming a single conserved diapause program and aims to distinguish shared regulatory features from lineage-specific adaptations while identifying priorities for future research under increasing environmental variability.

## 2. Review Methodology

A structured literature search was carried out to identify research on crustacean diapause from environmental, physiological, molecular, ecological, and evolutionary perspectives. The literature search primarily covered studies published between 2015 and 2025, matching the timeframe of the studies included in this review. Electronic bibliographic databases and academic search platforms (Web of Science Core Collection, Scopus, PubMed, and Google Scholar) used during manuscript preparation were searched, along with manual screening of the reference lists of pertinent primary research articles and review papers, to identify relevant studies. Studies were included if they provided evidence related to diapause induction, maintenance, or termination; environmental regulation; molecular and physiological mechanisms; metabolic adaptation; dormant-stage viability; or ecological and evolutionary consequences. Earlier foundational studies were included when necessary to provide essential mechanistic or conceptual context. The final literature search was conducted in late July 2026, and the evidence was synthesized narratively and thematically across environmental, physiological, molecular, ecological, and evolutionary dimensions.

Search terms were used individually and in combination and included terms related to crustacean diapause and dormancy, such as “crustacean diapause,” “crustacean dormancy,” “resting eggs,” “cysts,” “*Artemia* diapause,” “*Daphnia* diapause,” “copepod diapause,” “*Calanus* diapause,” and “*Neocalanus* diapause.” Mechanistic and environmental terms such as “photoperiod,” “temperature,” “salinity,” “hypoxia,” “food limitation,” “gene expression,” “transcriptomics,” “proteomics,” “FoxO,” “heat shock proteins,” “hormonal regulation,” “epigenetic regulation,” “metabolic suppression,” “energy storage,” “climate change,” and “population dynamics” were combined with these core search terms where appropriate. A representative Boolean search strategy was: (“crustacean” OR “Crustacea” OR “*Artemia*” OR “*Daphnia*” OR “Cladocera” OR “copepod” OR “*Calanus*” OR “*Neocalanus*”) AND (“diapause” OR “dormancy” OR “resting eggs” OR “cysts” OR “encysted embryos” OR “developmental arrest”) AND (“photoperiod” OR “temperature” OR “salinity” OR “hypoxia” OR “food limitation” OR “gene expression” OR “transcriptomics” OR “proteomics” OR “FoxO” OR “heat shock proteins” OR “hormonal regulation” OR “epigenetic regulation” OR “metabolic suppression”). Additional articles were also located by screening the reference lists of relevant reviews and primary research articles to identify studies not retrieved during the initial search.

Studies were considered eligible if they reported data directly relevant to crustacean diapause, including induction, maintenance, or termination of diapause; dormant-stage formation or viability; environmental regulation; physiological or metabolic adaptation; gene-expression or molecular regulation; hormonal or epigenetic mechanisms; ecological consequences; or evolutionary implications. Experimental studies, field observations, transcriptomic, proteomic, epigenomic, physiological, and comparative studies were considered. Studies that did not report on diapause or diapause-related dormancy in crustaceans provided insufficient information for interpretation, or only reported unrelated stress responses without demonstrating a link to diapause were excluded. Because the amount and type of evidence varied substantially among crustacean groups, studies were interpreted according to the type and strength of supporting evidence. Evidence strength was assessed qualitatively based on the nature of the evidence, with greater weight given to functional experiments and physiological studies demonstrating a direct association with diapause, while transcriptomic, gene-expression, and other omics-based findings without functional validation were interpreted as correlative evidence. Where possible, results from direct experimental manipulation were separated from associations identified through transcriptomic, proteomic, gene-expression, observational, or comparative studies. Extrapolated mechanisms were interpreted with caution when applied to crustacean lineages in which they had not been directly investigated. The tables were compiled from studies that met the above relevance criteria. The information extracted was organized under the following headings, as appropriate for each table: taxon/species, diapause life stage, habitat, environmental cue, diapause duration, physiological or metabolic adaptation, candidate molecular pathway or regulator, ecological consequence, and supporting reference. Where the evidence was associative, indirect, or inferred rather than functionally validated, this was indicated in the text or table as appropriate. Figures were created as conceptual frameworks representing relationships repeatedly reported in the selected literature, rather than as representations of any single pathway that has been experimentally validated.

## 3. Life-Stage Diversity and Ecological Significance of Diapause

Diapause can occur at different life stages in crustaceans, including embryonic, larval, juvenile, and adult stages, depending on the ecology, habitat stability, and reproductive strategy of the species [10,11]. This life-stage diversity shows that diapause is not a single uniform process, but a flexible adaptive strategy shaped by environmental pressures and evolutionary history [12,13]. Embryonic diapause is common in freshwater and hypersaline taxa such as *Daphnia*, *Artemia*, and *Boeckella*. These taxa can form dormant propagules, such as cysts or resting eggs, which allow survival under unfavorable conditions such as desiccation, salinity fluctuations, and temperature extremes [14]. These dormant stages are especially critical in hypersaline lakes, temporary ponds, and other environments with highly variable conditions. By contrast, marine copepods, e.g., *Calanus* and *Neocalanus*, tend to undergo diapause at the juvenile or adult stages. In these taxa, diapause is associated with metabolic depression, lipid accumulation, seasonal vertical migration, and synchronization of life cycles with phytoplankton availability and predation pressure. This contrast between embryonic diapause in branchiopods and copepodite-stage diapause in copepods highlights an important ecological trade-off. Resting eggs and cysts promote long-term persistence and recolonization after habitat disturbance, whereas copepod diapause mainly supports seasonal survival and reproductive timing in marine food webs. Therefore, diapause mechanisms are shaped not only by physiological constraints but also by habitat predictability, reproductive mode, and energy-storage strategy [11,15]. Variation in life-history traits, habitat characteristics, and environmental cues is reflected in the diversity of diapause strategies among crustaceans. An overview of these differences is provided in Table 1.

Ecologically, diapause is important because in taxa that produces dormant propagules, it contributes to dormant egg-bank formation, maintenance of genetic diversity, and rapid recolonization following environmental disturbance. In marine ecosystems, diapause in copepods helps synchronize emergence with plankton blooms, thereby supporting recruitment success and energy transfer across trophic levels [17,18]. Thus, diapause functions as a multi-scale adaptive strategy that contributes to cellular protection, life-history regulation, population persistence, and ecosystem processes.

## 4. Regulation and Physiological Dynamics of Diapause

### 4.1. Induction of Diapause

In crustaceans, environmental conditions act as cues for diapause induction, but their effects depend on species-specific life histories, developmental stages, and internal physiological states. Photoperiod, temperature, salinity, oxygen availability, and food limitation are among the major factors associated with the regulation of entry into diapause, although their relative importance differs among species and habitats [16,19]. In *Daphnia*, controlled environmental studies indicate that shortened day length and low food availability can promote the production of diapausing eggs, whereas in *Artemia*, experimental manipulation of culture conditions has shown that high salinity combined with photoperiodic and thermal cues can favor cyst formation [20]. In marine copepods, i.e., *Calanus* and *Neocalanus*, diapause induction is primarily associated with seasonal environmental cues such as declining food availability and temperature, as indicated by observational and physiological studies. These environmental conditions are accompanied by internal physiological and energetic changes, including lipid accumulation and alterations in physiological state, which may contribute to the initiation and maintenance of diapause. These species may enter juvenile or adult dormancy after accumulating sufficient energy reserves, a pattern interpreted as an adaptation that conserves energy until environmental conditions again favor development and reproduction [21,22,23]. This differs from embryonic diapause in branchiopods, which is generally interpreted as a long-term survival strategy in unstable or temporary habitats.

Importantly, diapause is rarely explained by a single environmental cue. Instead, experimental and comparative evidence indicates that multiple environmental signals can interact to influence the timing, depth, and duration of dormancy. For example, salinity, temperature, and photoperiod have been experimentally examined in combination and can modify cyst formation in *Artemia*, while food limitation and seasonal light changes have been reported to influence diapause responses in freshwater taxa [16,19,20]. The results presented here are consistent with a multi-cue model of diapause regulation, but the relative importance and causal significance of each cue remain incompletely understood in many crustacean groups. Thus, the notion that climate-driven disruption of environmental cues may lead to phenological mismatches is best viewed not as a generality across all crustaceans, but as an emerging ecological inference [22,24]. Molecular studies of diapause-related physiological responses, based on available transcriptomic, gene-expression, and physiological data, indicate that both neuroendocrine and gene-regulatory pathways are involved in translating environmental information into physiological responses.

Seasonal gene-expression patterns, clock-related pathways, lipid metabolism, and stress-response mechanisms have been associated with diapause timing and dormancy regulation in copepods and branchiopods [9,25,26]. Because much of this evidence is associative rather than based on direct functional manipulation, these pathways should be regarded as candidate components of diapause regulation rather than universally validated causal mechanisms. This regulatory integration is proposed to contribute to phenotypic plasticity by allowing crustaceans to adjust diapause timing and dormancy intensity according to local environmental variability [20,22].

### 4.2. Maintenance and Duration of Diapause

Diapause is maintained via coordinated metabolic depression, energy conservation and increased stress tolerance after induction [26,27]. Metabolic suppression reduces energy demand and limits cellular damage during prolonged dormancy. In some crustaceans, metabolic activity may decline to a small fraction of the active state, allowing stored lipids and carbohydrates to support survival when feeding and development are suspended [28,29]. Energy-storage strategies differ among taxa and reflect habitat-specific requirements. Some marine copepods store huge amounts of lipids before diapause, and these reserves support in overwintering, respiration, reproductive preparation, and post-diapause recovery [26,29,30]. Conversely, *Artemia* cysts depending upon stored triacylglycerols, glycogen, and protective molecules, steadily mobilized during early reactivation and embryonic dormancy. The difference between these species showed diapause maintenance depending on ecology and metabolic physiology [27]. Cellular protection is also essential for maintaining viability during dormancy. Antioxidant systems help limit oxidative damage by controlling reactive oxygen species, whereas molecular chaperones, including small heat shock proteins and HSP70, contribute to protein stabilization, prevention of protein aggregation, and maintenance of protein function under stress. Cryoprotectants and subsequent solutes protect membranes and macromolecules during desiccation, salinity stress, and temperature fluctuation [8,14].

Maintenance of cell integrity and restrain development, dormant stages allowed by these mechanisms. The duration of diapause varies widely among crustaceans. *Daphnia* ephippial eggs may remain viable for long periods in sediment egg banks, whereas copepod diapause is usually synchronized with seasonal cycles and may last for several months [31,32]. Such variation is associated with an important ecological difference: long term embryonic dormancy allows persistence in unpredictable or temporary habitats, whereas seasonal diapause in marine copepods allows synchronization of their life cycle with food availability. Environmental fluctuations can also modify dormancy depth and duration, indicating that diapause maintenance is a plastic process rather than a fixed physiological state [27,33]. The characteristic examples of diapause duration and correlated metabolic/molecular adaptations of crustacean taxa are summarized in Table 2.

### 4.3. Reactivation/Termination from Diapause

Reactivation from diapause occurs when environmental and physiological conditions become permissive for renewed development, feeding, or reproduction. Diapause termination refers to the release from the dormant state, whereas post-diapause reactivation or hatching refers to the subsequent resumption of development or emergence from the dormant stage. Diapause termination can be influenced by changes in temperature, photoperiod, oxygen availability, habitat conditions, salinity, and food availability, although the relevant cues and their relative importance differ among taxa [37,38]. Freshwater species may respond to habitat refilling together with suitable temperature and light conditions, whereas marine copepods often resume development in association with seasonal changes in hydrography and food availability that favor subsequent growth and reproduction [39,40].

Physiologically, the transition from diapause to active development involves the resumption of metabolic and biosynthetic activities, including mobilization of lipid or carbohydrate reserves, activation of mitochondria, reinitiation of transcription and translation, and/or the resumption of developmental or cell-cycle processes. The strength of evidence, however, varies considerably for the molecular pathways that underlie this transition. In *Artemia*, transcriptomic and integrated ATAC-seq/RNA-seq analyses have revealed changes in signaling pathways, chromatin accessibility, metabolism, stress-response pathways, and developmental regulation during the termination and reactivation of embryonic diapause [9,24,39]. The association with the reactivation process in these studies is compelling, but no single candidate pathway has yet been shown, on its own, to be causally necessary for diapause termination. Likewise, transcriptomic studies of *Neocalanus flemingeri* emerging from diapause and during post-diapause development have identified coordinated changes in developmental programs, stress-response genes, energy metabolism, and protein synthesis and turnover [35,36] that, again, must be interpreted as associative evidence until their functional significance is confirmed through targeted experimental manipulation.

There is limited functional evidence. For instance, knockdown of HSP70 in *Artemia franciscana* has demonstrated a role for this molecular chaperone in cyst viability and stress tolerance [14] and has thus provided stronger causal evidence for the protective function of HSP70 during dormancy than is available for many candidates signaling pathways. However, this does not prove that HSP70 is directly involved in diapause termination. Thus, antioxidant pathways, chaperone systems, FoxO-associated signaling pathways, and other mechanisms identified primarily through transcriptomic, proteomic, or gene-expression studies should be considered candidate pathways contributing to reactivation unless a causal role in diapause termination has been demonstrated through targeted perturbation experiments. Ecologically, the timing of reactivation is critical. Premature emergence may expose individuals to food limitation or unfavorable environmental conditions, whereas delayed emergence may reduce growth or reproductive opportunities. The timing of hatching or emergence can therefore become synchronized with habitat refilling, phytoplankton blooms, and other favorable ecological conditions [39,40]. Therefore, the regulation and molecular mechanisms of diapause termination should be considered a coordinated physiological process supported by different levels of evidence, ranging from omics-based associations to comparatively few direct functional studies.

## 5. Genetic and Molecular Regulation of Diapause in Crustaceans

### 5.1. Gene Expression During Diapause

Developmental arrest, metabolic suppression, and stress tolerance are tightly linked to diapause, which is regulated by interacting gene-regulatory networks connecting environmental sensing with developmental and physiological responses [41]. Transcriptomic studies in *Artemia*, *Daphnia*, and copepods have identified diapause-associated changes in the expression of genes involved in protein protection, antioxidant defense, lipid and carbohydrate metabolism, cell-cycle control, and developmental signaling [2,7,34]. However, these genes and pathways have varying levels of supporting evidence and should not be considered to have equivalent mechanistic roles. Genes identified through differential-expression studies provide evidence of an association with diapause, whereas genes studied through targeted perturbation, such as RNA interference or other functional manipulation, provide stronger evidence for a causal contribution to diapause regulation. A third category consists of genes or pathways suggested primarily by sequence homology, pathway enrichment, in silico reconstruction, or comparisons with other taxa, and these should be considered hypothetical or candidate regulators until experimentally validated.

Molecular chaperones are among the most consistently reported diapause-associated gene products. Small heat shock proteins and HSP70-family members are associated with protein stabilization, prevention of protein aggregation, and maintenance of cellular homeostasis during dormancy [8,41]. Functional RNAi experiments in *Artemia franciscana* demonstrate that the regulation of diapause-associated molecular chaperones contributes to stress tolerance in dormant embryos [41]. In contrast, numerous genes associated with heat-shock or general stress responses have been identified in *Daphnia*, but these genes have been characterized largely on the basis of differential expression or broader gene-expression profiles [2,8,42]. Thus, even within well-studied stress-protection pathways, the strength of evidence differs among species and individual genes.

Transcription factors are also important candidate components of diapause regulation. Metabolic depression, antioxidant responses, lipid metabolism, and developmental arrest have been associated with heat shock factors, FoxO-associated signaling, and nuclear-receptor pathways. However, direct functional support is uneven. For example, In *Artemia*, *Hsf1* has been manipulated experimentally, whereas insulin-like peptide/FoxO components proposed to regulate diapause in *Calanus finmarchicus* are supported mainly by transcriptomic analyses and in silico reconstruction, with limited direct experimental validation [41,43]. FoxO-associated pathways are of particular interest because they can connect nutrient sensing with energy conservation and stress resistance, but their role as conserved regulators of crustacean diapause remains hypothetical in several taxa. Embryonic diapause in branchiopods is commonly associated with strong cellular-protection and developmental-arrest responses, whereas copepod diapause is associated with changes in lipid metabolism, seasonal development, and reproductive timing [7,34,35,36]. Many genes proposed as regulators have, however, been identified as diapause-associated candidates primarily through transcriptomic profiling in these groups and should therefore be regarded as putative regulatory candidates rather than functionally validated regulators. Post-transcriptional regulation may provide an additional level of control during diapause. Alternative splicing and small RNAs, including microRNAs, can modify gene expression in response to environmental and developmental signals. The functional support for the two miRNAs in *Artemia*, miR-100 and miR-34, is stronger because their involvement in cell-cycle regulation during diapause entry has been confirmed experimentally [44]. By contrast, small-RNA-mediated mechanisms reported in several other crustaceans are supported mainly by expression profiling or comparative evidence and therefore require further functional validation.

Although advances have been made, the mechanisms that mediate the processes from upstream signals such as photoperiod, temperature, and salinity to the initiation of specific transcriptional programs remain poorly understood. This gap reflects several limitations. First, many candidate genes and pathways have been identified primarily through differential-expression or other omics analyses, but without subsequent functional validation. Second, molecular information is still restricted to a relatively small number of well-studied taxa and developmental stages, making comparisons among crustacean lineages difficult. Third, few studies have investigated the complete regulatory sequence from environmental cue perception to downstream transcriptional changes and the resulting measurable diapause phenotype within the same experimental system. Future studies should therefore focus on targeted functional manipulation of candidate genes, including RNA interference or gene editing where feasible, as well as time-resolved and tissue-specific analyses and comparative experiments conducted under controlled environmental conditions. A question that remains unanswered is whether the recurring sets of genes associated with diapause are evolutionarily conserved across crustaceans or whether similar pathways have been recruited independently in response to comparable environmental pressures. A summary of representative genes, transcription factors, small RNAs, their proposed functions, and the level of evidence supporting their involvement in diapause is provided in Table 3.

### 5.2. Hormonal and Epigenetic Regulation

Available evidence for hormonal and epigenetic control of crustacean diapause is mostly based on transcriptomic, gene-expression, epigenomic, and comparative studies, and only a few regulators have been directly functionally validated. Thus, the pathways, epigenetic modifications, and small-RNA-mediated mechanisms described below should be interpreted according to the strength of the supporting evidence, with experimentally validated mechanisms distinguished from those that remain associative or hypothetical.

**Figure 2:**
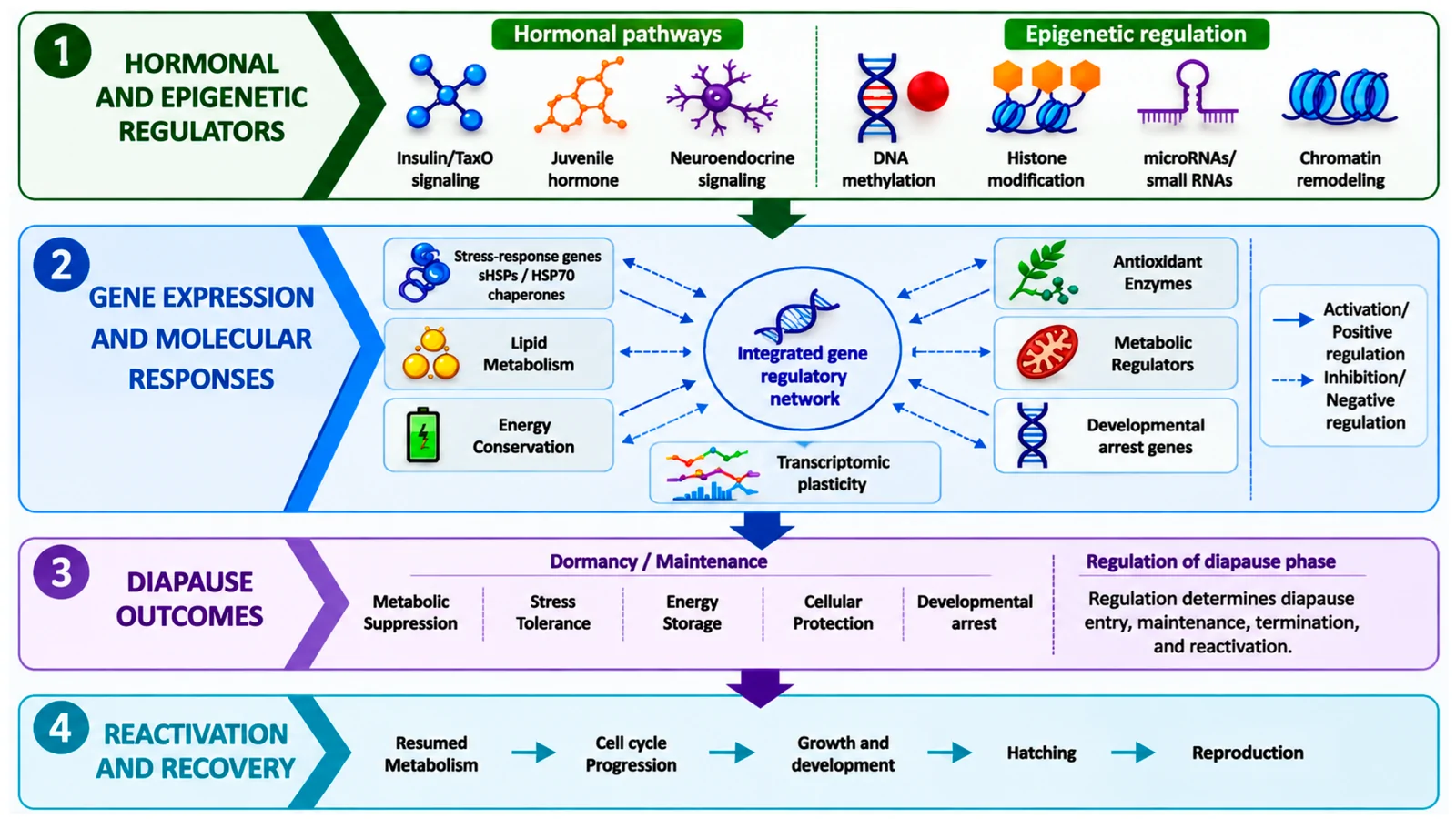
Conceptual framework illustrates the integrated regulatory mechanisms and physiological outcomes of diapause in crustaceans. (1) Hormonal and epigenetic pathways regulate (2) gene-expression networks involved in stress responses, metabolism, energy conservation, antioxidant defense, cellular protection, and developmental arrest. These processes support (3) diapause maintenance through metabolic suppression, stress tolerance, energy storage, and cellular protection, followed by (4) reactivation involving resumed metabolism, cell-cycle progression, growth, development, hatching, and reproduction. Solid arrows indicate proposed directional relationships, whereas dashed arrows indicate potential indirect or inhibitory interactions. The framework is comparative, as mechanisms vary among taxa, developmental stages, and habitats; omics-based associations require functional validation to establish causality.

During diapause, hormonal pathways act as a bridge between environmental cues and the body’s metabolic responses. Energy conservation, stress resistance, and survival during adverse environmental conditions are mediated by several pathways that cooperate to regulate other pathways, such as insulin/FoxO signaling, antioxidant defense pathways, nutrient sensing pathways, and developmental arrest pathways [10,46]. Reduced insulin-like signaling can activate FoxO-mediated transcriptional programs that promote energy conservation and stress resistance, allowing dormant individuals to maintain cellular stability under prolonged unfavorable conditions [47]. The juvenile hormone related pathways play an important role in the regulation of diapause, particularly in the suppression of reproduction, arrest of embryo development, and formation of cysts or resting eggs under environmental stress. In crustaceans, methyl farnesoate functions as a juvenile-hormone-like regulator and is associated with developmental and reproductive processes, while ecdysteroid signaling also contributes to developmental transitions [48,49]. However, hormonal roles appear to vary among crustacean groups and life stages. Hormonal regulation is more directly linked to developmental arrest and dormant propagule formation in embryonic diapause, but it may also be involved in lipid metabolism, reproductive timing, and seasonal life-cycle regulation in juvenile or adult diapause [9,50]. The epigenetic mechanisms are involved in the additional level of regulation that enables balance between stability and flexibility during diapause. DNA methylation, histone modification and chromatin remodeling are processes that impact gene expression and contribute to the establishment and maintenance of the transcriptional state of dormancy [6,51]. The epigenetic processes could play a role in long-term developmental arrest and in the process of responding to environmental cues that initiate the process of development. Small RNAs, particularly microRNAs (miRNAs) and piRNAs (piwi-interacting RNAs), also play a role in this regulation by regulating the expression of genes that are involved in stress tolerance, metabolism and development, thereby allowing flexible and reversible adaptation to environmental changes [51,52]. The integrated relationships among hormonal pathways, epigenetic regulation, gene-expression responses, diapause maintenance, and post-diapause recovery are summarized in Figure 2.

However, the role of hormones or epigenetic regulation of crustacean diapause is still not well understood. The majority of studies focus on transcriptomic or epigenomic associations, and only a few studies have directly validated candidate hormones, transcription factors, and epigenetic marks. The future line of research should take into account the use of an integrative approach combining endocrine assays, epigenomic profiling, small-RNA sequencing and gene-functional approaches to better understand the interaction of hormonal and epigenetic mechanisms, both within and across species, life stages and environments [6,9]. Representative hormonal and epigenetic regulators found in crustacean diapause along with their suggested physiological roles are summarize in Table 4.

Insulin/FoxO signaling, methyl farnesoate, and epigenetic regulators have been implicated in the regulation of diapause in several crustacean taxa, although the strength and nature of the supporting evidence vary among pathways and species. Most current studies are based on transcriptomic or gene-expression analyses, and relatively few use functional approaches such as gene knockdown, gene editing, or endocrine manipulation to establish causal relationships. Thus, whether these pathways represent lineage-specific adaptations to particular ecological conditions or constitute conserved regulatory mechanisms across crustaceans remains unclear. Distinguishing conserved regulatory mechanisms from taxon-specific adaptations will require comparative studies integrating functional genomics, endocrinology, and physiology across a broader range of crustacean taxa.

### 5.3. Metabolic Control and Energy Storage

Metabolic regulation is an important component in diapause maintenance as dormant crustaceans must maintain cellular function over lengthy periods of time while conserving energy [10,55]. During diapause, metabolic depression reduces the demand for ATP by lowering activity in energy-consuming pathways, including the tricarboxylic acid cycle and oxidative phosphorylation. This reduction not only conserves stored energy but also limits the production of reactive oxygen species, thereby decreasing cellular damage during dormancy [56]. Energy-storage strategies differ among crustacean groups. In marine copepods like *Calanus* and *Neocalanus*, the lipids can make up a significant part of the body mass and play the primary role in sustaining them over multiple months of diapause [45,57,58]. In *Calanus finmarchicus*, lipid reserves support overwintering, delayed development, and later reproductive activity, while lipid metabolism is sensitive to environmental conditions and diapause timing [21,26,56]. Unlike many live organisms, *Artemia* cysts are equipped with stored carbohydrates, trehalose associated protective compounds and other stored materials to help maintain the dormant state of the embryo and ensure the supply of resources for early development when the cysts are reactivated. These contrasting energy-storage strategies likely reflect adaptation to different ecological constraints rather than fundamentally different diapause mechanisms. Marine copepods experience prolonged overwintering in the water column and therefore depended on energy-dense lipid reserves to sustain extended dormancy and subsequent reproduction. In contrast, *Artemia* embryos must tolerate severe desiccation, osmotic stress, and prolonged encystment, making carbohydrates such as trehalose particularly advantageous because they function not only as energy reserves but also as protectants of proteins and cellular membranes. Thus, while metabolic suppression appears to be a conserved hallmark of crustacean diapause, the biochemical substrates supporting dormancy have diversified in response to species-specific habitats and life-history strategies. Protective metabolites are important for successful diapause. Desiccation, salinity stress and temperature fluctuations damage proteins, membranes and cellular structures, which is prevented by adding compounds like trehalose, glycerol, antioxidants, and other compatible solutes [13,59]. The level and length of metabolic suppression is not stable but depends on temperature, salinity, oxygen availability and life history of the species [21,27]. Overall, metabolic regulation during diapause involves a trade-off between energy conservation, stress resistance, and the ability to resume development and reproduction once favorable conditions return [10,26].

## 6. Environmental Cues and Their Interactions in Diapause Induction and Termination

**Figure 3:**
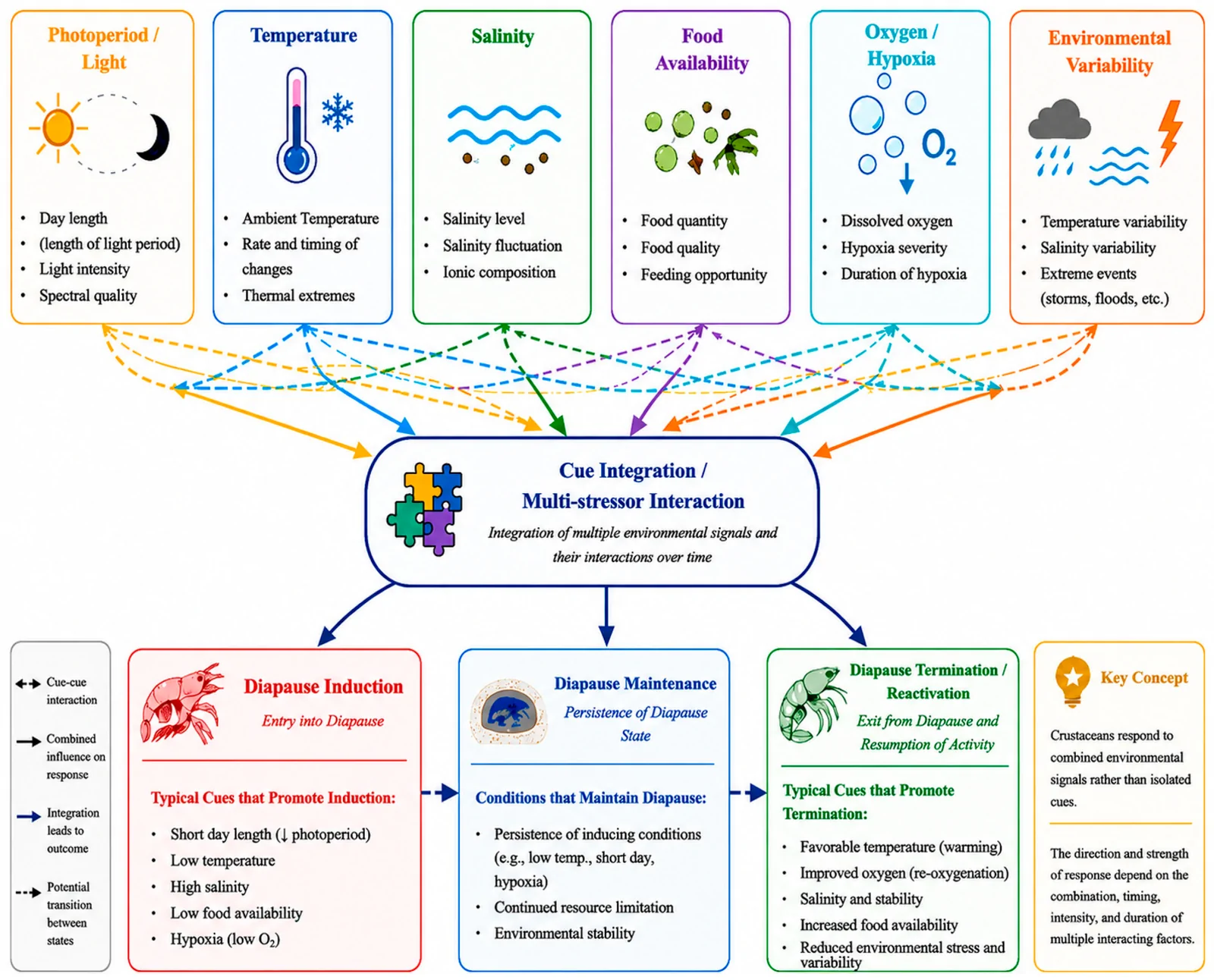
Environmental cue interactions regulating diapause induction and termination in crustaceans. The solid arrows show combined influences on diapause responses and dashed arrows show possible transitions between the environmental states. The colours of arrows indicate the different environmental cues displayed in the upper panels. Response direction and response level are determined by the combination, timing, intensity, and duration of the multiple interacting environmental factors.

In crustaceans, diapause regulation is most often multifactorial rather than controlled by a single factor. These external cues photoperiod, temperature, salinity, oxygen availability, and food conditions provide information about current and/or anticipated habitat suitability, but their effects depend on life stage, energetic state, reproductive status, and species-specific environmental sensitivity. These signals are thought to be integrated through interacting sensory and physiological pathways, such as circadian and photoperiodic pathways, neuroendocrine and hormonal signaling, nutrient- and energy-sensing pathways, and downstream transcriptional and metabolic responses [7,17,18,19,42,43,45]. The following regulatory mechanisms are proposed to translate combinations of environmental cues into developmental arrest, metabolic suppression, energy storage, stress protection, and reactivation. However, the relative significance of the various cues and the molecular sequence through which they are integrated remain incompletely understood and vary among crustacean taxa. The relationships in Figure 3 are therefore conceptual and represent an integration of proposed cue-integration mechanisms, rather than a single, universally validated regulatory pathway.

### 6.1. Photoperiod and Light Cues

Photoperiod is a critical signal for diapause regulation and a crucial indicator of season changes in environmental conditions [20,50]. In *Daphnia*, short photoperiods can stimulate ephippial egg production, while genetic differences in photoperiodic response may influence diapause timing and help maintain population diversity [60]. The reproductive strategies in *Artemia*, such as the production of cysts and hatching performance, are regulated by the interplay of photoperiod, salinity and temperature [16,19]. Circadian, neuroendocrine and molecular pathways are involved in the conversion of photoperiodic signals to physiological responses. In *Daphnia magna*, circadian clock genes are differentially expressed during diapause induction, suggesting that day-length perception relates to dormancy-related developmental regulation [50,61]. These pathways may link external light conditions with metabolic suppression, developmental arrest, and stress-tolerance mechanisms. However, light regulation is not limited to day length alone. A variety of factors affecting the light (intensity, spectral quality, duration, and timing), as well as the diapause response, are important for aquatic organisms living in an environment subject to varying light conditions and can affect the hatching responses of such organisms [38,62].

Evidence of relationships between photoperiod and other environmental factors comes from a variety of experimental designs and thus must be assessed according to the strength of the supporting evidence. Relatively direct evidence for the modification of diapause-related or dormant-stage responses to combinations of photoperiod, temperature, salinity, and light conditions comes from controlled environmental-manipulation experiments carried out with *Daphnia* and *Artemia* [17,18,19,63]. Conclusions about cue integration in marine copepods are made primarily from field observations, seasonal comparisons, and physiological or transcriptomic associations linking diapause timing with hydrographic conditions, food availability, seasonal light regimes, and internal physiological state [7,20]. The relationships derived from these observations are ecologically informative but do not by themselves establish the causal contribution of any single cue. Future studies should thus utilize multifactorial experimental designs with the simultaneous manipulation of several environmental variables, integrated with transcriptomic, epigenomic, metabolomic, endocrine, and physiological investigations. These approaches will help clarify how combinations of environmental signals are processed and translated into diapause phenotypes and whether cue-integration mechanisms are broadly shared or lineage-specific among crustaceans.

New research reveals that the relationship between photoperiod and light intensity, between light intensity and the spectral composition, and between the spectral composition and temperature and salinity are not independent but are interrelated and probably occur together. For example, light conditions can affect diapause progression and hatching in *Daphnia magna*, while photoperiod, temperature, and salinity jointly affect *Artemia* reproductive patterns and cyst-related outcomes [19,63]. These findings indicate that crustaceans may use complex photic information to improve the timing of diapause entry and reactivation. However, molecular mechanisms of interactions between different light signals, temperature, salinity and feed cues, remain poorly understood [60,64]. The significance of photoperiod seems to vary widely between different groups of crustaceans. Freshwater taxa (e.g., *Daphnia*) tend to use photoperiod as a predictable time of year indicator for anticipating a habitat change, while the marine copepods may combine photoperiod with food availability, hydrographic conditions and endogenous seasonal rhythms. This variation is suggestive that the hierarchy of environmental cues has developed not only because of phylogeny but also as a result of the predictability of the habitat. Yet, the mechanisms involved in cue integration are still not well understood, and comparative studies are needed in multiple crustacean groups.

### 6.2. Temperature and Seasonal Variability

Temperature is a factor in the regulation of diapause, both as an inducing and terminating factor, depending upon species, developmental stage and environment [65,66]. In many freshwater crustaceans, declining autumn temperature promotes ephippial egg production, whereas increasing temperature during favorable seasons may support hatching and reactivation [37,66]. Marine copepods regulate entry into, maintenance of, vertical distribution and emergence from diapause in response to interactions among temperature, seasonal food supply, hydrography and photoperiod [23,67]. Temperature is seldom the only factor influencing diapause. In *Artemia*, cyst formation is generally promoted by stressful environmental conditions, particularly high salinity, short photoperiod, and low temperature, whereas active reproduction is favored under more favorable environmental conditions, including suitable temperature and non-stressful salinity. Thus, the reproductive mode depends on the combined effects and intensity of environmental cues rather than on salinity alone [16,19,68]. In marine copepods, diapause termination is often synchronized with seasonal warming and phytoplankton blooms, allowing emergence to coincide with improved feeding conditions and reproductive opportunity [18,67]. Temperature also influences diapause depth and duration. Lower temperatures can extend dormancy by reducing metabolic rate and conserving energy reserves, while higher temperatures may shorten diapause or increase basal metabolic costs during reactivation [37,65]. Crustaceans might be able to adapt locally due to the variation in temperature thresholds within populations, though it remains to be seen how much protection this plasticity can offer at the end of a rapid environmental change [18,69]. Temperature is well known as an important regulator of diapause, but this regulation is highly context dependent and is generally not studied as the sole factor. Intra and inter-species differences indicate that temperature may be a factor that affects other environmental factors such as photoperiod, food availability, and salinity rather than being a standalone trigger. This complexity complicates predictions of climate-change impacts because identical thermal conditions may produce different diapause responses depending on ecological context, developmental stage, and local adaptation. Future studies should therefore adopt multifactorial experimental designs that better reflect natural environmental variability.

### 6.3. Salinity and Other Stressors

In crustaceans, salinity is a principal factor in inducing diapause in those inhabiting estuarine, hypersaline and halophile habitats. The high salinity in *Artemia* causes cyst formation and lowers moderate and declining salinity causes the development of naupliar and hatching of cysts [5]. This response reflects an adaptive strategy in which cyst production increases when osmotic stress signals unfavorable conditions for active development [68]. Salinity often interacts with other environmental cues rather than acting independently. In *Artemia*, salinity frequently interacts with photoperiod and temperature, demonstrating that diapause induction depends on integrated environmental signaling [16,19]. Similar interactions are likely to occur in other crustaceans facing fluctuating oxygen, food, or pollution, or drying habitats [70,71]. Other factors (hypoxia, contaminants, habitat change) may also affect the timing, depth and success of diapause. These factors may affect the quality of resting eggs or cysts, reducing hatching success, desiccation tolerance, and long-term viability [14,72]. Therefore, diapause outcomes depend not only on whether dormant stages are produced, but also on their physiological quality and ability to survive until favorable conditions return. These stressor-induced modifications may have an impact on recruitment, population stability, and ecosystem function [70,71]. The effects of critical environmental conditions on initiation and termination of diapause are summarized for representative species of crustaceans in Table 5.

### 6.4. Multiple Stressor Interactions

Diapause is caused by a combination of environmental factors, not just a single cue, in natural systems. The effects of interactions among photoperiod, temperature, salinity, oxygen and food availability can result in non-linear species-specific responses that affect diapause induction, depth, duration and termination [20,76]. This multi-stressor regulation allows crustaceans to adjust dormancy timing more precisely, but it also increases vulnerability when environmental cues become mismatched [77,78]. In *Artemia*, diapause responses are strongly influenced by interactions among salinity, temperature, and photoperiod, demonstrating that cyst formation depends on integrated environmental signaling rather than individual cues alone [4,5]. In *Daphnia*, ephippial egg production is influenced by short photoperiod and decreasing temperature, but these effects can be modified by food limitation, population density, salinity stress, or predator-related cues [20,76,79]. These examples show that diapause responses depend on cue combinations, threshold effects, and ecological context. Understanding multi-stressor interactions is especially important under climate change. Warming, altered salinity, hypoxia, pollution, and shifts in food availability may disrupt diapause timing, reduce dormant-stage survival, or create mismatches between emergence and resource availability [71,80]. Thus, future models should incorporate molecular regulatory mechanisms, physiological states and influence of environmental signals on diapause success [77,78].

Although substantial progress has been made in identifying environmental cues associated with diapause, most experimental studies manipulate only one or two variables under controlled laboratory conditions. Consequently, current understanding may underestimate the complexity of diapause regulation in natural ecosystems, where photoperiod, temperature, salinity, oxygen availability, food resources, and anthropogenic stressors fluctuate simultaneously. Integrating multifactorial experiments with field-based observations and molecular analyses will therefore be essential for developing predictive models of diapause responses under future environmental change.

## 7. Ecological and Evolutionary Implications of Diapause in Crustaceans

### 7.1. Diapause and Population Dynamics

Diapause is a basic mechanism that allows populations to persist in the face of environmental change, i.e., seasonal or unpredictable. Crustaceans can resist food scarcity, temperature changes, a lack of oxygen, habitat instability, and habitat desiccation because they produce long-lived dormant stages [31]. Genetic diversity is maintained and recovery of population is rapid in the case of *Daphnia* resting eggs and *Artemia* cysts in freshwater and hypersaline habitats [12]. Dormant egg banks function as biological reservoirs. They reduce the risk of local extinction during unfavorable periods and allow populations to recolonize habitats after drought, salinity stress, or other disturbances. It is especially critical in temporary ponds and fluctuating aquatic environments that experience a dynamic water level, where active populations can cease to exist while dormant propagules persist in pond sediments [40].

In marine copepods such as *Calanus finmarchicus* and *Neocalanus flemingeri*, diapause primarily functions to synchronize population dynamics with seasonal environmental cycles rather than to facilitate long-term egg banking in sediments. Emergence from diapause is frequently coordinated with the appearance of phytoplankton blooms, allowing individuals to resume feeding, development, and reproduction when resources are available [18]. By aligning development with favorable conditions, such synchronization enhances recruitment success and facilitates the flow of energy transfer across planktonic food webs [17,73]. Diapause also supports bet-hedging, where only part of a population resumes development while another fraction remains dormant. This strategy spreads survival risk across time and protects populations from unpredictable environmental failure [18,40]. However, environmental disturbances may thwart these benefits. With climate change and anthropogenic stressors, recruitment failures and cascading ecological effects could change because of changes in the hatching and emergence schedule and the availability of food [81]. The population-level importance of diapause differs among crustacean taxa, with dormant stages supporting persistence, seasonal synchronization, recolonization, and bet-hedging under fluctuating environmental conditions (Table 6).

### 7.2. Evolutionary Perspective

Diapause is a survival mechanism which has evolved to maintain fitness of organism in unfavorable environments, seasonally, temporarily or unpredictably [10,83]. It is adaptive value varies by life stage, habitat stability, and reproductive strategy between crustaceans’ group. Embryonic diapause produces persistent resting eggs or cysts in *Daphnia* and *Artemia* which can remain in sediments for years, resulting in continued presence of those species and maintenance of genetic variation [31,84]. In marine copepods, diapause is often expressed at copepodite-stages and is closely linked with seasonal food availability, lipid storage, and reproductive timing [11,67].

Crustacean diapause shares several physiological and molecular features with diapause-like strategies in insects and nematodes, including metabolic suppression, enhanced stress tolerance, insulin/FoxO-associated signaling, hormone-mediated developmental control, and epigenetic regulation [10,83]. However, similar regulatory features, should not be taken as evidence of evolutionary conservation. Some might be homologous and inherited from common ancestry, while others could have been recruited independently under similar selective pressures, such as seasonal food limitation, temperature stress, or habitat unpredictability, in different lineages. These possibilities can only be distinguished with evidence of orthology, conserved pathway organization, comparable functional roles, and, where possible, experimental validation across phylogenetically distinct taxa. There is as yet insufficient evidence to determine whether diapause represents an evolutionarily conserved, integrated regulatory program across major invertebrate groups or whether similar physiological outcomes have arisen through largely independent molecular pathways. Though, crustaceans also have ecological adaptations, such as dormant egg bank in freshwater and a seasonally timed response to plankton blooms in marine conditions. A comparison of diapause and diapause-like evolutionary trends in major invertebrate groups is given in Table 7.

Habitat heterogeneity has likely shaped variation in diapause induction, duration, and cue sensitivity among crustacean species. This diversification of diapause strategies is also likely to be driven by multiple interacting evolutionary processes. Under varying degrees of environmental predictability, alternative timing thresholds, dormancy durations, and cue sensitivities may be advantageous, while bet-hedging may spread recruitment risk across time in highly variable habitats. Local adaptation can also generate population-specific responses to photoperiod, temperature, salinity, or food, while phenotypic plasticity can cause variation in diapause expression without underlying genetic differences among individuals. The tendency, timing, or quality of dormant-stage production may also be influenced by maternal and transgenerational effects, depending on parental environmental experience. Over longer evolutionary timescales, differences in reproductive strategy, developmental timing, energetic allocation, and habitat use may drive lineage-specific combinations of regulatory and physiological mechanisms. These processes are not mutually exclusive and are likely to act in concert to account for the substantial variation in diapause phenotypes observed among crustaceans [10,83,85].

Temporary pond species benefit from long-term dormant propagules and bet-hedging, whereas marine copepods benefit from precise seasonal timing and energy-storage strategies [85,86]. These evolutionary differences are crucial for understanding which crustaceans would be resilient to environmental change and which might be sensitive to the disruption of a seasonal cue, modification in their habitat, or mismatch to climate change [67,83]. Despite the apparent conservation of metabolic suppression, endocrine regulation, and stress-resistance pathways across many invertebrates, it remains uncertain whether these similarities reflect shared evolutionary ancestry or repeated convergent evolution driven by comparable environmental pressures. Comparative functional studies across crustaceans, insects, nematodes, and other dormant organisms will be necessary to distinguish universally conserved regulatory networks from lineage-specific adaptive innovations. Such comparative approaches could substantially improve our understanding of the evolutionary origins and diversification of diapause mechanisms.

### 7.3. Impact of Climate Change

Climate change has the potential to significantly change environmental elements involved in crustacean diapause regulation, such as temperature, photoperiod-temperature interaction, salinity, oxygen, and feeding. Warming may shorten dormancy duration, increase metabolic demand during diapause, and deplete stored energy reserves before favorable conditions return [27,69]. These changes may create phenological mismatches, where hatching or emergence no longer coincides with peak food availability or suitable environmental conditions [18,35]. Freshwater crustaceans may be especially susceptible to altering seasonal timing. Warming earlier than usual can modify resting egg hatching or juvenile development occur at wrong time, resulting in reducing recruitment if food availability and optimal environmental conditions have already passed [37,65]. In hypersaline and temporary aquatic systems, increased evaporation and salinity can affect dormant-stage production, hatching, and viability, as well as reduce the ability of dormant stages to respond to freshwater cues that regulate the timing and duration of dormancy [70,72]. Climate change is expected to intensify interactions among multiple environmental stressors, potentially reducing diapause success, dormant-stage viability, and population resilience in the crustacean taxa for which evidence is currently available [70,71]. Such disruptions may weaken population resilience, alter the persistence and diversity of dormant propagule banks where present, and affect trophic synchronization in marine and freshwater ecosystems. As climate change continues to alter aquatic environments, there is a clear need for integrated approaches that connect environmental cues with molecular responses, energy balance, survival during dormancy and population dynamics to better predict their effects on crustacean diapause [18,27].

Current projections of climate-change impacts on crustacean diapause remain constrained by limited long-term ecological datasets and a predominance of laboratory-based studies conducted under simplified environmental conditions. Consequently, the adaptive capacity of natural populations and the extent of evolutionary responses remain difficult to predict [87]. Future studies should be conducted using long-term ecological studies alongside functional genomics and transcriptomics, physiological measurements, and multifactorial experiments that simultaneously manipulate temperature, salinity, oxygen availability, food availability, and seasonal timing under environmentally realistic climate-change scenarios. These experiments should be integrated with field-derived environmental data to determine whether molecular and physiological responses correspond to the range and covariance of conditions experienced by natural populations [88]. Combining such datasets with demographic, phenological, and ecosystem-level simulation models could enable experimentally determined changes in diapause induction, duration, energy utilization, survival, diapause termination, and reactivation to be translated into predictions of recruitment and population persistence [89]. Model validation against long-term field observations would add further confidence to predictions across species and habitats. All of these strategies will provide a more mechanistic basis for understanding crustacean resilience under future climate variability.

## 8. Limitations and Future Outlook

Despite the significant advances made in the study of crustacean diapause, there are still some important limitations that constrain mechanistic interpretation and cross-taxon comparisons. These limitations can be grouped into data, taxonomic, methodological, functional-validation, and ecological-scaling gaps.

Data gaps. The information on crustacean diapause is far from consistent, and there is considerably more molecular, physiological, and ecological information available for a few well-studied species than for most other crustaceans. The available datasets also vary widely with respect to experimental design, developmental stage, environmental conditions, and analytical resolution, making direct comparisons between studies difficult. Therefore, more standardized and comparable data across species, life stages, and environmental conditions are required to distinguish broadly shared diapause-associated responses from context-specific responses.

Taxonomic gaps. Most current knowledge is focused on well-studied taxa such as *Artemia*, *Daphnia*, and marine copepods, whereas many other crustacean lineages remain poorly characterized. Groups of particular interest include Ostracoda and several lineages of Malacostraca, such as decapods, amphipods, and isopods, as well as other understudied crustacean groups for which molecular and functional information on dormancy remains limited. Although dormant or resting stages have been documented in ostracods, their molecular regulation is far less characterized than that of *Daphnia*, *Artemia*, or major copepod taxa. Broader taxonomic sampling across these phylogenetically and ecologically divergent groups would help determine whether recurrent diapause-associated processes reflect conserved mechanisms, lineage-specific innovations, convergent responses to similar environmental pressures, or biases arising from the current concentration of research on a few taxa. It would also provide a stronger foundation for interpreting the evolutionary diversification of crustacean dormancy in relation to differences in habitat, developmental stage, reproductive strategy, and life history. Therefore, the extent to which the molecular, physiological, and ecological mechanisms observed in these intensively studied taxa are conserved across Crustacea remains uncertain, and some may instead represent lineage-specific adaptations. Comparative studies that include a wider phylogenetic and ecological range are needed to distinguish potentially conserved regulatory features from taxon-specific diapause strategies.

Methodological gaps. Recent transcriptomic, physiological, and ecological research has yielded valuable insights into the regulation of diapause, but such studies often differ in experimental design and analytical approaches, which makes it difficult to compare results across studies. Furthermore, many laboratory investigations examine a single environmental factor under controlled conditions that may not reflect the combined variation in photoperiod, temperature, salinity, oxygen availability, and food availability that occurs in natural habitats. Standardized experimental designs, multifactorial environmental manipulations, and complementary molecular and physiological measurements should therefore be incorporated into future studies.

Functional-validation gaps. One major limitation is that many candidate genes, signaling pathways, hormonal regulators, and epigenetic mechanisms have been identified based on transcriptomic, proteomic, or gene-expression associations, but relatively few have been directly tested to establish their causal roles. Greater focus should therefore be given to functional approaches such as gene knockdown, gene editing, controlled endocrine manipulation, and other targeted experimental strategies capable of establishing direct links between candidate regulatory pathways and diapause phenotypes. These studies are crucial to distinguish between regulators that have been functionally demonstrated to influence diapause and those that are only associated with diapause.

Ecological-scaling gaps. Another major limitation is the insufficient integration of molecular, physiological, population-level, and ecosystem-level responses. Research tends to concentrate on individual levels of biological organization, such as molecular regulation, organismal physiology, or population dynamics, rather than on the interactions among these levels. Furthermore, the effects of multiple environmental factors on diapause performance, dormant-stage survival, recruitment, and population persistence remain poorly understood under ecologically realistic conditions. Combining mechanistic experiments, long-term ecological monitoring, comparative field studies, and predictive modelling will therefore be important for clarifying how diapause contributes to population resilience under environmental variability and climate change.

Overall, a combination of interdisciplinary approaches integrating molecular biology, functional genomics, physiology, ecology, and evolutionary biology across diverse crustacean taxa and environmental conditions will be critical for future advances. This integration will help distinguish potentially conserved from lineage-specific diapause mechanisms and improve predictions of how crustacean populations may respond to future environmental change.

## 9. Conclusions

This review identifies four broad conclusions regarding diapause in crustaceans. First, diapause is a complex process controlled through interactions among several environmental factors, including photoperiod, temperature, salinity, oxygen availability, and food supply, and the relative importance of these factors differs among taxa, habitats, developmental stages, and physiological states. Second, metabolic suppression, developmental arrest, cellular protection, antioxidant defense, and energy conservation emerge as recurring features of crustacean diapause; however, whether their underlying molecular and hormonal mechanisms are evolutionarily conserved across crustacean lineages remains unresolved. Third, diapause strategies vary considerably across life-history types, with dormant cysts and resting eggs in taxa like *Artemia* and *Daphnia* being closely related to long-term persistence and recolonization, whereas juvenile and/or adult diapause in marine copepods is closely linked to reproductive timing, lipid-based energy storage, and seasonal synchronization. Fourth, recent transcriptomic, epigenomic, and other omics studies have revealed numerous genes and regulatory pathways associated with diapause, although many of these relationships remain associative due to limited direct functional validation.

Future research should thus focus on functional validation of candidate genes, endocrine pathways, and epigenetic modulators; broaden comparative studies beyond the well-studied taxa that have dominated research to date; and include multifactorial experiments that better simulate natural combinations of environmental cues. The integration of these approaches with long-term ecological monitoring, population-level assessments, and predictive modelling will be critical to understanding the role of diapause in crustacean resilience to changes in temperature, salinity, oxygen availability, food supply, and seasonal timing. This integrative research will provide a stronger mechanistic basis for predicting how crustacean populations and aquatic ecosystems may respond to increasing environmental variability.

## Figures and Tables

**Table 1:** Representative crustacean species, diapause stage, habitat type, and major environmental triggers.

Species	Diapause Stage	Habitat Type	Major Environmental Triggers	References
*Artemia franciscana*	Embryonic cyst	Hypersaline lakes	High salinity, photoperiod, temperature	[16]
*Daphnia pulex*	Embryonic ephippial egg	Freshwater ponds	Short photoperiod, declining temperature, food limitation	[1]
*Boeckella similis*	Embryonic resting egg	Temporary freshwater pools	Photoperiod, temperature, habitat drying	[12]
*Calanus finmarchicus*	Copepodite stage V (CV; pre-adult)	Marine pelagic environment	Food limitation, temperature, seasonal photoperiod	[7]
*Neocalanus flemingeri*	Adult female; CIV in the large form	Marine pelagic environment	Seasonal cycles, temperature, food availability	[17]
*Cyclops strenuus*	CIV/CV copepodites; pre-fertilized/fertilized adult female	Freshwater systems	Photoperiod, temperature, seasonal habitat change	[12]

**Table 2:** Diapause duration and associated metabolic and molecular adaptations in representative crustaceans.

Species	Approximate Diapause Duration	Metabolic/Physiological Adaptations	Major Molecular Mechanisms/Evidence	Evidence Basis/Limitation	References
*Artemia franciscana*	Months to years	Profound metabolic depression and developmental arrest; trehalose- and LEA-protein-associated protection of dormant cysts	Small heat shock proteins, particularly p26, artemin, and LEA proteins associated with cellular protection and stress tolerance	Biochemical and functional evidence is available for several protective proteins; the cited synthesis does not establish FoxO signaling as a general mechanism in this species.	[13]
*Daphnia pulex*	Prolonged dormancy in sediment resting-egg banks	Resting-egg production under food limitation; altered fatty-acid biosynthesis and carbohydrate metabolism associated with resting-egg formation	Candidate genes identified by differential-expression analysis before resting-egg production	Controlled food manipulation combined with RNA-seq; candidate genes remain associative and have not yet been functionally validated.	[2,32]
*Calanus finmarchicus*	Several months	Large lipid reserves supporting seasonal overwintering; deep-water diapause and reduced activity	Diapause-associated transcriptional regulation of metabolic, developmental, and stress-related processes	Supported mainly by field observations and transcriptomic analyses; individual candidate pathways have generally not been functionally validated.	[7,34]
*Neocalanus flemingeri*	Approximately 5–7 months	Lipid accumulation before diapause, markedly reduced metabolic activity during dormancy, and subsequent activation of the reproductive program	Diapause-associated suppression of protein synthesis/turnover and regulation of stress-response genes; post-diapause activation of energy-metabolism and developmental pathways	Supported by physiological measurements and transcriptomic profiling; these associations should not be interpreted as direct functional validation of individual regulatory genes.	[35,36]
*Boeckella poppei*	Prolonged embryonic dormancy; dormant embryos have been reported to persist for very long periods in Antarctic sediments	Developmental arrest, protective multilayered cyst wall, preservation of yolk reserves, and post-diapause yolk utilization	No specific molecular regulatory pathway was directly tested in the cited study	Evidence is primarily ultrastructural and developmental; molecular mechanisms should therefore not be extrapolated from other crustaceans.	[12]

**Table 3:** Representative genes, proteins and regulatory components associated with diapause in crustaceans and the level of evidence supporting their proposed roles. Differential-expression, transcriptomic, proteomic, and computational associations are distinguished from direct functional manipulation. Absence of functional validation indicates that a proposed pathway should not be interpreted as causally demonstrated.

Species/Taxon	Representative Genes, Proteins or Pathways	Regulatory Component	Proposed Diapause-Related Role	Level of Evidence/Limitation	References
*Artemia franciscana*	*Hsf1*; p26, ArHsp21, ArHsp22 and artemin; miR-100 and miR-34	*Hsf1*; diapause-associated miRNAs	Stress tolerance, molecular-chaperone regulation, and cell-cycle arrest	Functional validation/experimental manipulation. *Hsf1* has been examined by RNAi, and miR-100/miR-34 have functional evidence for regulation of cell-cycle-associated targets. These findings provide relatively strong mechanistic support for specific components but do not establish a universal *Artemia* diapause network.	[41,44]
*Daphnia* spp.	Resting-egg-associated differentially expressed genes; *HSP70*-family genes; circadian-clock-associated genes	Stress-response and clock-related transcriptional regulation	Resting-egg production, stress response, and photoperiod-associated developmental regulation	Predominantly transcriptomic/gene-expression evidence. Resting-egg-related candidates identified by RNA-seq remain largely associative; clock-related studies provide experimental evidence for photoperiodic responses, but most individual diapause genes have not been functionally validated.	[2,8,42,45]
*Calanus finmarchicus*	Metabolism-, development-, stress-, and lipid-related genes; insulin-like peptide/FOXO components	Candidate FOXO/nuclear-receptor and metabolic regulatory systems	Seasonal metabolic suppression, lipid utilization, development, and diapause timing	Transcriptomic and in silico evidence. Annual transcriptome studies show strong diapause-associated expression patterns, while the ILP/FOXO system has been reconstructed as a candidate regulatory pathway; direct gene-specific functional validation remains lacking.	[7,34,43]
*Neocalanus flemingeri*	Protein-synthesis and turnover genes; metabolic and stress-response genes; developmental regulators	Nuclear-receptor-, forkhead-, and transcription-associated candidates	Dormancy maintenance, energy conservation, and post-diapause reactivation	Transcriptomic evidence. Differential expression during diapause emergence and post-diapause development supports association, but individual regulatory genes have not been directly functionally validated.	[35,36]
*Boeckella poppei*	No specific diapause-regulatory gene was directly tested in the cited study	Not established	Developmental and structural characteristics of dormant/post-diapause embryos	Ultrastructural/developmental evidence only. The cited study does not support assignment of FoxO, HSF, trehalose-synthase, miRNA, or other specific molecular regulators. Molecular mechanisms should therefore not be inferred from other crustacean taxa.	[12]

**Table 4:** Hormonal and epigenetic/post-transcriptional regulators associated with diapause or diapause-related dormancy in representative crustaceans. Evidence is classified according to whether the reported relationship is experimentally supported, functionally validated, transcriptomically associated, or indirectly inferred. Associative omics evidence should not be interpreted as demonstrating causal regulation.

Species	Hormonal Regulators	Epigenetic/Post-Transcriptional Regulators	Proposed Role in Diapause	Evidence Basis/Limitation	References
*Artemia franciscana*	Direct evidence for a specific endocrine master regulator of diapause remains limited; signaling pathways involved in diapause termination/reactivation have been identified as candidates	Changes in chromatin accessibility during embryonic reactivation; miR-100 and miR-34	miR-100 and miR-34 contribute to cell-cycle arrest during diapause entry, whereas changes in chromatin accessibility accompany transcriptional reactivation after diapause	Functional evidence is available for diapause-associated miRNAs; ATAC-seq/RNA-seq provides epigenomic and transcriptional association rather than direct causal validation of individual chromatin regulators	[24,44]
*Daphnia pulex*	Methyl farnesoate and associated JHAMT–Met/RXR signaling	DNA methylation and histone modifications have been characterized in *D. pulex*, but their direct roles in diapause remain unresolved	Methyl farnesoate influences the reproductive switch, including male and ephippia production; a direct causal role for the reported epigenetic marks in diapause has not been established	Methyl-farnesoate effects are supported by experimental exposure studies; available epigenomic evidence is indirect with respect to diapause	[6,53]
*Calanus finmarchicus*	Insulin-like peptide/FOXO signaling components proposed as candidate regulators of seasonal pre-adult diapause	Possible involvement of clock-associated epigenetic regulation has been inferred from annual transcriptomic patterns, but direct diapause-specific epigenetic manipulation is lacking	Potential coordination of nutrient sensing, metabolic regulation, and seasonal diapause timing	The ILP/FOXO system was reconstructed largely from transcriptomic/in silico evidence and proposed as a possible contributor to diapause; functional validation is lacking	[7,43]
*Neocalanus flemingeri*	Nuclear hormone receptor-, RXR-, and forkhead-box-associated transcripts	Chromatin-silencing genes and histone lysine methyltransferase-associated transcripts	Candidate involvement in transcriptional suppression during dormancy and transcriptional reactivation during emergence from diapause	Supported primarily by differential gene-expression/transcriptomic evidence; direct endocrine or epigenetic functional validation has not yet been demonstrated	[36,54]
*Boeckella poppei*	No specific diapause-related hormonal regulator was directly examined in the cited study	No diapause-specific epigenetic or small-RNA regulator was directly examined	No causal hormonal or epigenetic mechanism can presently be assigned from the available cited evidence	Available evidence is primarily ultrastructural and developmental; molecular mechanisms should not be extrapolated from *Artemia*, *Daphnia*, or copepods without direct evidence	[12]

**Table 5:** Effects of environmental cues on diapause induction and termination in crustaceans.

Environmental Cue	Effect on Diapause Induction	Effect on Diapause Termination	Species Examples/Notes	References
Photoperiod/light	Short or decreasing day length can promote diapause entry	Longer photoperiod may support reactivation in some species	Important in *Daphnia* and *Artemia*	[20]
Temperature	Low or declining temperature may induce diapause	Rising temperature can stimulate emergence and metabolic reactivation	Important in *Calanus finmarchicus* and *Neocalanus flemingeri*	[27]
Salinity	High salinity can promote cyst formation	Moderate or decreasing salinity may favor hatching	Especially important in *Artemia franciscana*	[16]
Food availability	Food limitation can stimulate diapause in some taxa	Improved food supply may support reactivation and development	Reported in *Daphnia, Boeckella*, and copepods	[73]
Oxygen/hypoxia	Low oxygen may deepen dormancy or delay development	Normoxia may support reactivation and post-diapause recovery	Relevant to *Artemia* and *Calanoid* copepods	[74,75]

**Table 6:** Population-level effects of diapause in representative crustaceans.

Species/Taxa	Diapause Type	Population-Level Effects	Notes/Examples	References
*Daphnia pulex*	Embryonic diapause/ephippial eggs	Population persistence, rapid recolonization, bet-hedging	Resting eggs survive drought, food limitation, and unfavorable pond conditions	[20]
*Artemia franciscana*	Embryonic cysts	Maintenance of genetic diversity and population resilience	Cysts form dormant egg banks in hypersaline lakes	[13]
*Calanus finmarchicus*	Adult diapause	Seasonal population synchrony and survival during low-food periods	Emergence is synchronized with phytoplankton blooms	[18]
*Neocalanus flemingeri*	copepodite-stage diapause	Life-cycle synchronization and seasonal population stability	Lipid reserves support months-long dormancy	[82]
*Boeckella similis*	Embryonic/larval diapause	Rapid recovery after temporary pool refilling and bet-hedging	Dormant stages survive variable freshwater conditions	[31]

**Table 7:** Comparative overview of diapause and diapause-like evolutionary strategies across invertebrate groups.

Taxa	Diapause Type	Evolutionary Strategy/Adaptive Significance	Example Species	References
*Crustaceans*	Embryonic, larval, juvenile, or adult diapause	Bet-hedging, dormant egg-bank formation, seasonal synchronization, survival under environmental variability	*Artemia*, *Daphnia*, *Calanus*	[10]
*Insects*	Embryonic, pupal, or adult diapause	Metabolic suppression, hormonal regulation, photoperiodic timing, survival during seasonal stress	*Aedes*, *Drosophila*, *Leptinotarsa*	[59]
*Nematodes*	Dauer larva	Developmental arrest, stress resistance, dispersal, survival under resource limitation	*Caenorhabditis elegans*	[10]

## Data Availability

No new datasets were generated or analyzed during the current study.

## Abbreviations

The following abbreviations are used in this manuscript:

HSP	Heat shock protein
FoxO	Forkhead box O (transcription factor)
piRNAs	piwi-interacting RNAs
miRNA	MicroRNA
DNA	Deoxyribonucleic acid
RNA	Ribonucleic acid
ATP	Adenosine triphosphate
CIV	Copepodite Stage IV (fourth copepodite stage)
CV	Copepodite Stage V (fifth copepodite stage)
